# Effect of Ondansetron on Intra-procedural Nausea and Vomiting in Patients Undergoing Transcatheter Aortic Valve Replacement With Conscious Sedation: A Before-and-After Study

**DOI:** 10.7759/cureus.91070

**Published:** 2025-08-26

**Authors:** Hiroki Anezaki, Shingo Kawashima, Kensuke Suzuki, Tetsuro Kimura, Yoshiki Nakajima, Hiroyuki Kinoshita

**Affiliations:** 1 Department of Anesthesiology and Intensive Care Medicine, Hamamatsu University School of Medicine, Hamamatsu, JPN; 2 Department of Anesthesiology, Seirei Mikatahara General Hospital, Hamamatsu, JPN; 3 Department of Anesthesiology, Toyohashi Heart Center, Toyohashi, JPN

**Keywords:** conscious sedation, nausea, ondansetron, transcatheter aortic valve replacement, vomiting

## Abstract

Purpose: Sedation has advantages for patients receiving transcatheter aortic valve replacement (TAVR) compared with general anesthesia. However, the beneficial effect of an antiemetic agent on intra-procedural nausea and vomiting (IPNV) remains unclear. Therefore, we aimed to evaluate whether ondansetron reduces IPNV in patients undergoing transfemoral TAVR with conscious sedation.

Methods: We conducted a single-center retrospective study, analyzing outcomes of 100 patients undergoing TAVR before (Before group, n=48) and after (After group, n=52) ondansetron administration. Conscious sedation was initiated with a bolus of 0.7 μg/kg and subsequent 0.1-0.4 μg/kg/h IV dexmedetomidine, supplemented with IV remifentanil and IV propofol to achieve a bispectral index <90 with a Richmond Agitation-Sedation Scale of -2 to -1. Only the After group received ondansetron 4 mg IV at the beginning of sedation. All patients underwent an ultrasound-guided right or left fascia iliaca block using 30 mL of 0.185% ropivacaine, depending on the sheath insertion side. The primary endpoint was IPNV incidence during TAVR procedures, with statistical significance set at P<0.05.

Results: Groups were well balanced, with no significant differences in aortic stenosis severity, cardiac function, or comorbidities affecting heart function. The After group showed a significantly lower incidence of IPNV (0% vs. 22.9%, P<0.001 using the χ^2^ test). No differences were observed in intra-operative vasopressor use, and no cases of suspected aspiration occurred.

Conclusions: Ondansetron prevented IPNV in transfemoral TAVR patients with conscious sedation. These results suggest that pretreatment with ondansetron may be a useful strategy for reducing aspiration risk in procedures requiring conscious sedation, including transfemoral TAVR.

## Introduction

The application of a sedation technique to patients with aortic stenosis undergoing transfemoral transcatheter aortic valve replacement (TAVR) began less than a decade ago [1-3]. A large-scale analysis using more than 120,000 TAVR procedures documented the increased use of sedation during TAVR up to 64% in the United States, indicating that the role of sedation technique in TAVR procedures is increasingly critical [4]. However, deep sedation frequently causes respiratory adverse events, including the need for airway maneuvers to secure the airway and bag-mask ventilation [1]. A previous study demonstrated a shorter length of stay and lower in-hospital and 30-day mortality in patients undergoing TAVR with conscious sedation than general anesthesia [2]. Therefore, many clinicians currently favor conscious, but not deep, sedation during transfemoral TAVR.

Procedural deep sedation causes nausea and vomiting in less than 10% of cases [5]. In addition, hypotension causes nausea and vomiting, which was precluded by a vasoconstrictor infusion [6]. Accordingly, a previous paper argued that cardiovascular derangements, including hypotension and opioid use during TAVR under sedation, induce intra-procedural nausea and vomiting (IPNV), resulting in an increased risk of aspiration in the population [7]. However, previous studies evaluating the feasibility of transfemoral TAVR have never paid attention to IPNV and prophylactic antiemetic use.

Ondansetron, a 5-hydroxytryptamine type 3 receptor antagonist, is safe and valuable for preventing and treating postoperative emesis as a single use [8,9]. Indeed, a recent large-scale meta-analysis found high-certainty evidence that ondansetron is one of the antiemetic agents that reduces postoperative nausea and vomiting (PONV) [7]. However, the beneficial effect of the agent on IPNV during conscious sedation has been unclear.

Therefore, we aimed to evaluate whether ondansetron reduces IPNV in patients undergoing transfemoral TAVR with conscious sedation in the current study. To verify our hypothesis, we performed the study in a single-center retrospective fashion and measured the outcomes of 100 patients undergoing TAVR before and after ondansetron installation in one of our affiliated hospitals.

This article was previously presented as a meeting abstract at the 2025 International Anesthesia Research Society Annual Meeting on March 20-23, 2025.

## Materials and methods

We performed the current study in a single-center retrospective fashion using data between March 2021 and December 2023 at the Department of Anesthesiology, Toyohashi Heart Center, Toyohashi, Japan. Ethical approval for this study (#240301) was provided by the Ethical Committee of Toyohashi Heart Center on May 28, 2024. In addition, this study was registered in the University Hospital Medical Information Network Clinical Trials Registry (UMIN000056446). The procedures in the current study followed the “Declaration of Helsinki” and the ethical standards of the responsible committee on human experimentation. Patients before (Before group, n=48) and after (After group, n=52) ondansetron installation above 20 years old with severe aortic stenosis who underwent transfemoral Sapien 3^TM^ (Edwards Lifesciences Corp., Irvine, CA, USA) or Evolut^TM^ (Medtronic, Minneapolis, MN, USA) replacement were included in the study. Cardiologists determined whether each patient was eligible for transfemoral TAVR and which prosthesis was adequate for treatment. Patients with a predicted difficult airway, severe pulmonary arterial hypertension (systolic pulmonary arterial pressure >60 mmHg), former/current alcohol or drug abuse, neurocognitive/neurodegenerative disease or psychiatric disorder, or use of antidepressants/sedatives were excluded. The secondary exclusion criteria were perioperative emergency conversion from sedation to general anesthesia and the need for cardiopulmonary resuscitation.

Treatment and measurement during TAVR

None of the patients had premedication, whereas all prescribed medications, except for angiotensin receptor antagonists and angiotensin-converting enzyme inhibitors, were continued until the morning of TAVR. Patients undergoing transfemoral TAVR usually receive conscious sedation during the procedure at Toyohashi Heart Center. Conscious sedation was defined according to the American Society of Anesthesiologists as follows [10]. It is a drug-induced depression of consciousness. Patients respond purposefully to verbal commands, either alone or by light tactile stimulation; no interventions are required to maintain a patent airway, and spontaneous ventilation is adequate. The depth of sedation was monitored using bispectral analysis (BISTM; Covidien Japan Inc., Tokyo, Japan) throughout sedation and valve replacement.

All patients had the ultrasound-guided right or left fascia iliaca block (FIB) using 30 mL of 0.185% ropivacaine, depending on which side the expandable sheath was inserted, according to our previous study [11]. All FIB procedures were completed within five minutes. We added infiltration analgesia with 1% lidocaine, 13 mL, to the procedural regions. In addition, we instructed patients that they had to inform us of their pain experience during TAVR, if any, since we would administer them a rescue regimen for the occasion. Conscious sedation for each patient under 100% face mask oxygen supplementation (6 L/min) was initiated with 0.7 µg/kg and subsequent 0.1-0.4 µg/kg/hr of IV dexmedetomidine. The sedation was supplemented with 0.03 µg/kg/min of IV remifentanil and 0.3 mg/kg/hr of IV propofol to achieve the BIS value less than 90 with the Richmond Agitation-Sedation Scale (RASS) -2 to -1 [12]. As all patients’ sedation levels were set within RASS -2 to -1, they could notify the anesthesiologist in charge of their sedation at any time about their IPNV and painful condition during the TAVR procedure. Only the After group patients received ondansetron 4 mg IV before administering sedative medications at the onset of sedation, and any patients who suffered from IPNV received 10 mg of IV metoclopramide. We simultaneously administered 20 µg of IV remifentanil upon each patient’s pain notice during TAVR [11]. Invasive arterial pressure monitoring was applied, and a temporary pacemaker wire was inserted through the femoral vein for rapid ventricular pacing. Implantation was guided by fluoroscopy. All patients received 0.01 to 0.07 µg/kg/min of IV noradrenaline and 0.05 mg of IV phenylephrine several times to maintain a mean arterial pressure above 65 mmHg. All anesthetic management for TAVR procedures was performed solely by HK. We recorded hemodynamic and sedation parameters at the beginning of TAVR, the 15 minutes, 30 minutes, and at the end. The primary endpoint was the incidence of IPNV during TAVR procedures.

Statistical analysis

Statistical analysis was performed using IBM SPSS Statistics for Windows, Version 27 (Released 2021; IBM Corp., Armonk, New York, United States). Continuous variables are presented as the median and interquartile (25-75th percentile) range and were compared between groups using the Mann-Whitney U test. Categorical variables are presented as the number of cases with percentages and were compared between groups using the χ^2^ test. Differences were considered to be statistically significant at P<0.05. The power calculation was also done using IBM SPSS. A 22% difference in the incidence of IPNV gave 96% power to detect a significance level of α = 0.05 with a sample size of 52 in the current study.

## Results

We analyzed 103 patients scheduled for elective transfemoral TAVR under conscious sedation. Three patients were excluded because they did not meet our inclusion criteria; thus, the remaining 100 patients were enrolled into the before (Before group, n=48) and after (After group, n=52) ondansetron installation group.

As shown in Table 1, the enrolled patients' preoperative characteristics were comparable except for body mass index and hemoglobin levels, and the incidence of carotid stenosis and chronic obstructive pulmonary disease. All patients had symptoms of New York Heart Association (NYHA) class II or III. Groups were well balanced with no significant differences in aortic stenosis severity, cardiac function, and co-morbidity related to heart function (Table 2).

**Table 1:** Patients' preoperative characteristics. Data are shown as median (IQR). Categorical variables are given as the number of cases (%). BMI: body mass index; COPD: chronic obstructive pulmonary disease

	Before (n=48)	After (n=52)	P-value
Female patients	31 (64.6)	36 (69.2)	0.621
Age (years)	84 (80 to 87)	84 (80 to 88)	0.696
BMI (kg/m^2^)	23.1 (19.4 to 25.6)	20.4 (18.3 to 23.1)	0.019
Hypertension	30 (62.5)	33 (63.4)	0.921
Diabetes mellitus	16 (33.3)	14 (26.9)	0.485
Carotid stenosis	0 (0)	5 (9.6)	0.028
Prior stroke	13 (27.1)	22 (42.3)	0.111
Hemodialysis	9 (18.8)	7 (13.5)	0.471
COPD	22 (45.8)	13 (25.0)	0.029
Hemoglobin (g/dL)	12.0 (10.7 to 12.8)	10.7 (10.0 to 11.8)	0.002

**Table 2:** Patients' preoperative characteristics related to heart condition. Data are shown as median (IQR). Categorical variables are given as the number of cases (%). AR: aortic valve regurgitation; CABG: coronary artery bypass grafting; MR: mitral valve regurgitation; NYHA: New York Heart Association; PCI: percutaneous coronary intervention; TR: tricuspid valve regurgitation

	Before (n=48)	After (n=52)	P-value
NYHA (II/III) (n)	42/6	44/8	0.678
Aortic valve area (cm^2^)	0.73 (0.58 to 0.83)	0.67 (0.55 to 0.82)	0.152
Mean pressure gradient aortic valve (mmHg)	43 (35 to 50)	44 (36 to 54)	0.494
Maximum pressure gradient aortic valve (mmHg)	76 (69 to 88)	74 (67 to 92)	0.388
Left ventricle ejection fraction (%)	63.7 (51.8 to 68.8)	60.9 (55.0 to 65.2)	0.065
Left main trunk stenosis ≧50%	2 (4.2)	0 (0)	0.137
Proximal left anterior descending artery stenosis ≧70%	4 (8.3)	8 (15.4)	0.278
Moderate to severe AR	7 (8.3)	5 (9.6)	0.445
Moderate to severe MR	10 (20.8)	10 (19.2)	0.841
Moderate to severe TR	7 (14.6)	6 (11.5)	0.651
Prior myocardial infarction	3 (6.3)	2 (3.8)	0.582
Prior PCI	5 (10.4)	6 (11.5)	0.858
Prior CABG	1 (2.1)	2 (3.8)	0.606
Atrial fibrillation	14 (29.2)	7 (13.5)	0.054
Prior pacemaker	2 (4.2)	1 (1.9)	0.511

Procedure-related values are listed in Table 3. The After group patients showed significantly less incidence of IPNV (primary endpoint) compared with the Before group(0 vs. 22.9%); the difference was statistically significant (χ^2^(1) = 15.3, P < 0.001, φ = 0.347). However, as shown in Table 3, the After group documented a lower average propofol dose and lower mean arterial pressure values than the Before group. No differences between groups were observed for intraoperative vasopressor use during the procedures. No case was suspected of aspiration.

**Table 3:** Procedure-related values. Data are shown as median (IQR) for continuous variables. Categorical variables are given as the number of cases (%). HR: heart rate; MAP: mean arterial pressure; TAVR: transcatheter aortic valve replacement

	Before (n=48)	After (n=52)	P-value
Intra-procedure nausea and vomiting (n)	11 (22.9)	0 (0)	<0.001
Duration of sedation (min)	64 (52 to 74)	52 (45 to 60)	<0.001
Duration of TAVR (min)	48 (40 to 61)	40 (34 to 49)	0.003
Average remifentanil dose (ng/kg/min)	9.6 (7.0 to 12.4)	8.5 (7.3 to 11.3)	0.385
Average propofol dose (mg/kg/h)	0.09 (0.07 to 0.10)	0.08 (0.06 to 0.09)	0.022
Average dexmedetomidine dose (µg/kg/h)	1.03 (0.85 to 1.18)	1.09 (0.96 to 1.26)	0.054
MAP at TAVR beginning	108 (96 to 117)	87 (77 to 97)	<0.001
MAP at TAVR 15 min	87 (76 to 99)	82 (73 to 90)	0.04
MAP at TAVR 30 min	90 (81 to 101)	80 (67 to 89)	<0.001
MAP at TAVR end	81 (73 to 90)	70 (64 to 80)	<0.001
HR at TAVR beginning	68 (60 to 77)	60 (59 to 70)	0.032
HR at TAVR 15 min	63 (53 to 70)	60 (56 to 70)	0.675
HR at TAVR 30 min	65 (55 to 72)	65 (60 to 75)	0.161
HR at TAVR end	64 (60 to 70)	66 (60 to 75)	0.305

## Discussion

Our recent study indicates that conscious sedation combined with FIB in the sheath insertion side decreases opioid requirements while maintaining high patient satisfaction by the reduction of procedural pain [11]. In our preliminary observation, we found that some patients suffer from nausea during TAVR. A previous paper argued that cardiovascular derangements, including hypotension and opioid use during TAVR under sedation, induce IPNV, resulting in an increased risk of aspiration in the population [7]. However, previous studies evaluating the feasibility of transfemoral TAVR have never paid attention to IPNV and prophylactic antiemetic use. In the current study, no incidence of IPNV after IV ondansetron installation (0%), compared with before installation (22.9%), is somewhat surprising since procedural deep sedation is known to cause nausea and vomiting in up to 10% of the incidence [5]. Importantly, the group after IV ondansetron installation documented lower mean arterial pressure values than the group before IV ondansetron installation, whereas there were no differences between groups for intra-operative vasopressor use and average remifentanil dose during the procedures. Therefore, we can conclude that different cardiovascular derangements and opioid use in the current study did not affect the antiemetic effect of IV ondansetron. However, it is unclear whether IV ondansetron can prevent IPNV caused by severe and prolonged hypotension during the TAVR procedure, since we can treat nausea and vomiting caused by hypotension during spinal anesthesia using a vasoconstrictor infusion [6].

A recent large-scale meta-analysis has demonstrated high-certainty evidence that a 5-hydroxytryptamine type 3 receptor antagonist, ondansetron, is an antiemetic agent that reduces PONV [13]. The agent is effective as a single-use toward PONV since its antiemetic effect of a single dose persists for up to six hours, with three hours of the half-life [14,15]. Indeed, ondansetron reduces the PONV incidence by less than half in single use [16]. These support our results that ondansetron is also a valuable agent for reducing IPNV lasting less than several hours.

While several antiemetics are now available for clinical use worldwide, it remains unclear which drug is most effective. Weibel et al. implemented a network meta-analysis to evaluate the efficacy of various medications in preventing PONV after general anesthesia [17]. Indeed, 5-hydroxytryptamine type 3 receptor antagonists, dopamine D2 receptor antagonists, neurokinin 1 receptor antagonists, and corticosteroids equally prevent PONV by using a single dose, although the mechanistic insight into the antiemetic effect is different from each other [17]. Among these compounds, a 5-hydroxytryptamine type 3 receptor antagonist, ondansetron, and dopamine D2 receptor antagonists, droperidol and metoclopramide, are clinically available for perioperative use in Japan, although combinations of drugs must be more effective than a single agent used to prevent PONV. However, D2 receptor antagonists may cause a risk of extrapyramidal symptoms and QT prolongation in the electrocardiogram. Therefore, employing ondansetron as a single agent to prevent IPNV during TAVR procedures under conscious sedation seems reasonable.

We must mention some limitations in the current study. First, we performed the current study in a single-center retrospective fashion before and after ondansetron installation. The data from retrospective analysis might cause potential biases. However, since the study was conducted by a single anesthesiologist in a before-and-after design, we believe that the management approach and patient backgrounds were relatively consistent, minimizing potential bias. Second, in the current study, the surgery and anesthesia times were shorter in the After group with IV ondansetron. We cannot rule out the possibility that these results might affect the incidence of IPNV after ondansetron installation. Third, we did not examine the long-term outcomes in our study patients, whereas both groups of patients demonstrated similar uncomplicated admissions for TAVR without postprocedural nausea and vomiting. More extensive studies will be required to overcome the above limitations.

## Conclusions

To the best of our knowledge, our study is the first to demonstrate that ondansetron strongly prevents IPNV in patients undergoing transfemoral TAVR with conscious sedation. Our results suggest that pretreatment with ondansetron may be a strategy to avert aspiration during conscious sedation in procedures, including transfemoral TAVR. However, further prospective large-scale studies will be required to verify the ondansetron’s beneficial effect on patient care during conscious sedation.
